# Physician assessment of disease activity in JIA subtypes. Analysis of data extracted from electronic medical records

**DOI:** 10.1186/1546-0096-9-9

**Published:** 2011-04-14

**Authors:** Michael L Miller, Jason Ruprecht, Deli Wang, Ying Zhou, George Lales, Sean McKenna, Marisa Klein-Gitelman

**Affiliations:** 1 Division of Rheumatology, Department of Pediatrics; Feinberg School of Medicine, Northwestern University, Chicago, IL, USA; 2 Children's Memorial Research Center (CMRC), Children's Memorial Hospital, Chicago, IL, USA; 3 Department of Information Technology, CMRC, Feinberg School of Medicine, Northwestern University, Chicago, IL, USA; 4 Biostatistical Research Core, CMRC; Feinberg School of Medicine, Northwestern University, Chicago, IL, USA

## Abstract

**Objective:**

Although electronic medical records (EMRs) have facilitated care for children with juvenile idiopathic arthritis (JIA), analyses of treatment outcomes have required paper based or manually re-entered data. We have started EMR discrete data entry for JIA patient visits, including joint examination and global assessment, by physician and patient. In this preliminary study, we extracted data from the EMR to Xenobase™ (TransMed Systems, Inc., Cupertino, CA), an application permitting cohort analyses of the relationship between global assessment to joint examination and subtype.

**Methods:**

During clinic visits, data were entered into discrete fields in ambulatory visit forms in the EMR (EpicCare™, Epic Systems, Verona, WI). Data were extracted using Clarity Reports, then de-identified and uploaded for analyses to Xenobase™. Parameters included joint examination, ILAR diagnostic classification, physician global assessment, patient global assessment, and patient pain score. Data for a single visit for each of 160 patients over a 2 month period, beginning March, 2010, were analyzed.

**Results:**

In systemic JIA patients, strong correlations for physician global assessment were found with pain score, joint count and patient assessment. In contrast, physician assessment for patients with persistent oligoarticular and rheumatoid factor negative patients showed strong correlation with joint counts, but only moderate correlation with pain scores and patient global assessment. Conversely, for enthesitis patients, physician assessment correlated strongly with pain scores, and moderately with joint count and patient global assessment. Rheumatoid factor positive patients, the smallest group studied, showed moderate correlation for all three measures. Patient global assessment for systemic patients showed strong correlations with pain scores and joint count, similar to data for physician assessment. For polyarticular and enthesitis patients, correlation of patient global assessment with pain scores was strong. Moderate correlations were found between patient global assessment and joint count in oligoarticular and polyarticular patients.

**Conclusion:**

Data extraction from the EMR is feasible and useful to evaluate JIA patients for indicators of treatment responsiveness. In this pilot study, we found correlates for physician global assessment of arthritis differed, according to disease subtype. Further data extraction and analyses will determine if these findings can be confirmed, and will assess other outcome measures, compare longitudinal responses to treatment, and export extracted data to multi-center databases.

## Introduction

Juvenile idiopathic arthritis (JIA) is characterized by joint inflammation with onset at or before sixteen years of age [1]. Recent studies of biologic agents [2,3], have relied upon a core set of outcome measures, including components of physical examination, physician and patient global assessment, and pain scores [4]. Other disease status measures validated to measure remission [5] and define minimally active disease [6] have found that, despite current treatment, many children, including those with initial response, have disease recurrences, measured by remission criteria [7].

Physician global assessment is an important component of all these outcome measures, distinct from joint counts, patient pain assessment, and other components. Physician and patient global assessments have been used to define minimal disease activity [6]. Studies have not determined the extent to which physician global assessment correlates with joint counts or pain assessments, depending upon JIA subtype. The advent of the electronic medical record (EMR) permits data extraction to address this type of question more efficiently than permitted by a study of paper based records.

Since 2008, we have used an EMR to document all outpatient visits, resulting in more rapid and better organized access to medical information. We recently incorporated the ability to enter discrete data for all visits of children with juvenile idiopathic arthritis. In addition to extracting data in preparation for participation in multi-center studies, we have started to analyze extracted data concerning clinical status and assessment of JIA patients. In this preliminary study, we used EMR data extraction to study the relationship of physician global assessment to patient global assessment and pain scores in patients with different JIA subtypes.

## Patients and methods

### Patients

We studied data for all patients meeting ILAR diagnostic criteria for juvenile idiopathic arthritis [8] seen by M.M. and M.K.G. at Children's Memorial Hospital pediatric rheumatology clinics for 2 months starting March 2010. Patients in the Undifferentiated arthritis category were not included because of heterogeneous characteristics of these patients. In the other ILAR subtypes, 160 patients were seen.

### Demographic and disease characteristics

Age data (birth date, date of each visit) were extracted. Additional extractable disease characteristics consisted of diagnostic subtype, joint count (active joints, as defined in reference [4]), pain score, physician and patient global assessment. ANA, rheumatoid factor (RF), and B27 status had been tested within the first 4 months of diagnosis and available (as noted in parentheses) for the subgroups as follows: 18 systemic patients (18 ANA, 12 RF, 0 B27); 63 oligoarticular persistent patients (59 ANA, 43 RF, 21 B27); 6 oligoarticular extended patients (6 ANA, 5 RF, 2 B27); 40 RF negative polyarticular patients (38 ANA, 35 RF, 15 B27); 12 RF positive polyarticular patients (12 ANA, 12 RF, 6 B27); 3 psoriatic arthritis patients (3 ANA, 3 RF, 2 B27), 18 enthesitis patients (18 ANA, 15 RF, 15 B27). Per cent positive for each test is expressed as per cent of all patients in the subgroup.

### Physician and patient assessment

Physician global assessment of overall disease activity [4] used a scale from 0 to 10. Patient pain was rated on a Likert scale (with 0 = no pain and 10 = very severe pain) in response to the question "By giving a number between 0 and 10, with 0 being no pain and 10 being the worst possible pain, how much pain on average have you experienced from your arthritis over the past week?" Patient global assessment was rated on a Likert scale (with 0 = doing very poorly and 10 = doing very well) in response to the question "By giving a number between 0 and 10, with 0 being doing very poorly and 10 being doing very well, how have you experienced your arthritis in general over the past week? Include not only pain, but also how you feel about your arthritis, how having arthritis affects your getting along with family and friends, and how well you can move around." Of the 160 patients, 132 (83%) self reported pain and global assessments (13.0 ± 4.0 years). Of the other 28 patients (6.3 ± 2.7 years), mothers of 26 patients reported, and fathers reported for 2 patients.

### Data Entry into the Electronic Medical Record

**Figure 1:**
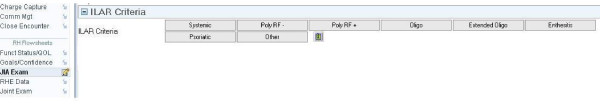
**Appearance of the flow sheet rows for diagnosis within the EpicCare™ EMR**. Entry of ILAR diagnosis is more accurate than can be accommodated by the ICD-9 system, implemented primarily for billing purposes. The ICD-9 system, for example, has no specific code for systemic JIA.

Since July 2008, all patient visits have been documented in an EMR (EpicCare™, Epic Systems, Verona, WI). Starting in 2010, a discrete data structure (called flow sheet rows in the Epic EMR) for JIA patients, named RHE modules, for JIA patients has been incorporated into the EMR, based upon the same data entry structure in use at Cincinnati Children's Hospital. All patients with JIA have disease subtype (Figure 1), joint examination and related clinical data entered as discrete data into these flow sheet rows, as part of routine care for each outpatient encounter. During the period of study, 3.75% of assessments were incomplete, reflecting adjustment of physican work flows to the new data entry method.

### Data Extraction from the Electronic Medical Record

**Figure 2:**
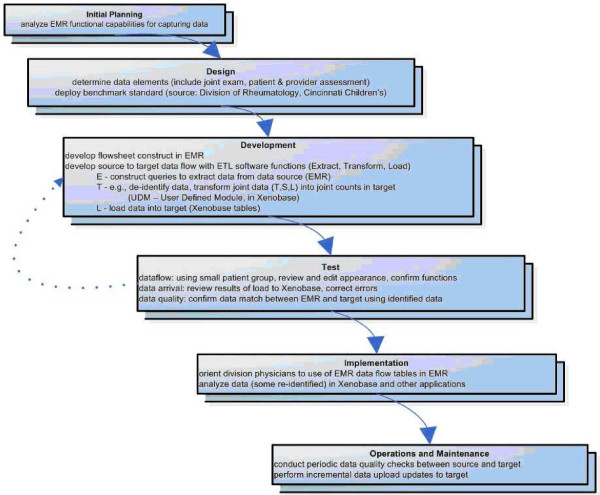
**System Development Lifecycle**. See Methods for explanation. Development initially affected appearance of the ambulatory EMR by adding forms for entry of specific values for the joint examination, physician and patient global assessment, and patient pain score. The ETL (extract, transform, load) process was implemented external to both the EMR source and analytic software target.

A System Development Lifecycle process was established by the Department of Information Technology, Children's Memorial Research Center for data extraction (Figure 2). Once flow sheet rows were established, an Extract, Transform, Load (ETL) procedure employed data queries that extracted relevant data, which was then de-identified prior to uploading to Xenobase™. Data is extracted each month for all RHE modules.

### Xenobase

**Figure 3:**
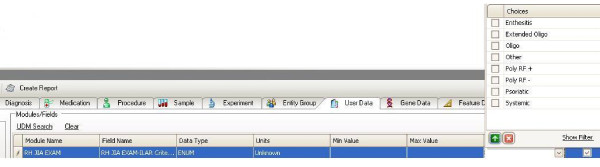
**Appearance of a data query window within the Xenobase™ analytic software**. Data queries, based upon JIA subtype, can be carried out on uploaded data extracted from the EMR.

Data was analyzed using the XenoBase-BioIntegration Suite (Xenobase™), an application residing in servers maintained by the Xenobase team, CMRC. Xenobase, developed in the Program of Translational Medicine at the Van Andel Research Institute, and available commercially (TransMed Systems, Inc., Cupertino, CA), provides a common interface for consolidating disparate clinical, preclinical and molecular data; it provides statistical and graphical data analytic functions (Figure 3). To provide patient privacy, Xenobase uses an offset of up to 45 days applied to all uploaded patient dates (including birthdates). With IRB approval, this offset was re-identified, permitting retrieval of legacy paper based data (dates of onset of symptoms and diagnosis for patients whose first visits antedated EMR usage) and data quality validation.

### Statistical methods

Descriptive statistics are presented as median (minimum, maximum) for continuous variables or frequency (percentage) for categorical variables. We used Spearmann Rank correlation coefficients to measure pair-wised correlations among physician assessment and patient pain score, global assessments, and joint counts for all subtypes except extended oligoarticular and psoriatic arthritis, due to the small numbers of patients in those groups. Correlations were considered strong for values >0.7, moderate for 0.4 - 0.7, and weak for <0.4. All statistical analyses were performed using SAS^® ^version 9.2 (SAS Institute, Inc., Cary, NC).

## Results

### Patients

We analyzed data extracted from 160 patients (Table 1). Racial and ethnic group distributions (Table 1) were notable for the paucity of African American and Asian patients seen with arthritis, relative to their representation among outpatients seen in specialty clinics at Children's Memorial Hospital (data not shown). In addition, Hispanic patients tended to have either oligoarticular or polyarticular disease, more than other subtypes.

**Table 1:** Demographic and disease characteristics

	Systemic	Oligo-p	Oligo-e	RF- poly	RF+ poly	Psoriatic	Enthesitis
N	18	63	6	40	12	3	18

Age (yrs)	10.7 (5.8, 19.9)	9.9 (2.6, 18.6)	15.3 (10.8, 21.1)	11.2 (3.5,1 9.6)	13.5 (8.9,20.1)	12.9 (8.6,17.6)	17.0 (9.7, 21.2)

Gender (F/M)	8/10	52/11	5/1	34/6	12/0	2/1	9/9

Onset age (yrs)	5.5 (0.9, 13.0)	3.7 (0.8, 15.8)	8.2 (1.6, 14.8)	4.5 (1,15.6)	11.2 (6.7,15.6)	7.3 (6.3,13.0)	10.3 (3.0, 15.6)

Diagnostic age (yrs)	6.1 (1.0, 13.3)	4.3 (1, 16.6)	9.2 (1.9, 15.3)	5.4 (1.3, 17.3)	11.8 (6.9,16.1)	7.4 (7.3,13.4)	13.2 (9.7, 18.4)

Duration from diagnosis (yrs)	0.1 (0,1.1)	0.3 (0, 10.0)	0.3 (0, 2.0)	0.4 (0.0, 5.4)	0.4 (0,1.0)	0.3 (0.2,1.0)	2.1 (0, 5.3)

ANA+ n (%)	4 (22%)	36 (57%)	4 (67%)	15 (37.5%)	7 (58%)	1 (33%)	9 (50%)

RF+ n (%)	0 (0%)	1 (1.5%)	1 (16.7%)	0 (0%)	12 (100%)	1 (33%)	1 ((5.6%)

B27+ n (%)	n/a	1 (1.5%)	1 (16.7%)	0 (0%)	0 (0%)	0 (0%)	6 (33%)

Race/Ethn.							

*White/non-Hispanic*	14	48	6	32	8	2	14

*Hispanic*	1	7		7	3		2

*African American*	2	1			1		1

*Asian*	1	3		1			

*Other*		4				1	1

Half of children with enthesitis related arthritis were ANA positive. Few patients with non-polyarticular disease were rheumatoid factor positive. One third of enthesitis related arthritis patients were B27 positive. Although no discernible differences among the subtypes were found for median scores for physician global assessment, patient global assessment, or patient pain score (Table 2), relationships between physician assessment and other parameters were found for particular disease subtypes, as noted below.

**Table 2:** Values of outcome measures

	Systemic	Oligo-p	Oligo-e	RF- poly	RF+ poly	Psoriatic	Enthesitis
**N**	**18**	**63**	**6**	**40**	**12**	**3**	**18**

Physician global assessment	3 (0,10)	2 (0,10)	4.5 (1,7)	3 (0,9)	2 (0,8)	0 (0,1)	3 (0,7)

Patient global assessment	8 (0,10)	9 (3,10)	5 (3,9)	8 (3,10)	8 (1,10)	9 (4,10)	7.75 (0, 10)

Patient pain score	2.5 (0,8)	1 (0,8)	5 (2,7)	2 (0,8)	2 (0,9.5)	0 (0,2)	4 (0,10)

Joint Count	0.5 (0,19)	0 (0,4)	7.5 (0,15)	1 (0,20)	2.5 (0,10)	0 (0,0)	0 (0,2)

Joint Count = 0 n (%)	9 (50%)	35 (56%)	2 (33%)	18 (45%)	5 (42%)	3 (100%)	10 (56%)

### Physician Global Assessment in JIA subtypes

Correlation of physician assessment with other outcome measures varied with JIA subtype (Table 3). In systemic JIA patients, strong correlations were found for pain scores, joint count and patient assessment. In contrast, physician assessment for patients with persistent oligoarticular and rheumatoid factor negative patients showed strong correlation with joint counts, but moderate correlation with pain scores and patient global assessment. Conversely, for enthesitis patients, physician global assessment correlated strongly with pain scores, and moderately with joint count (and, similarly to persistent oligoarticular and rheumatoid factor negative polyarticular patients, moderately with patient global assessment). Rheumatoid factor positive patients, the smallest group studied, showed only moderate correlation for all three measures.

**Table 3:** Spearman's correlations between Physician Global Assessment and other outcome measures in JIA subtypes

		Systemic	Oligo-p	RF- poly	RF+ poly	Enthesitis
*Physician Global Assessment vs*.						

	pain score	0.76	0.45	0.55	0.48	0.89

	joint count	0.77	0.84	0.84	0.43	0.69

	patient global assessment	-0.89	-0.62	-0.66	-0.48	-0.56

### Patient Global Assessment in JIA subtypes

Correlation of patient assessment with pain scores and joint counts also varied with JIA subtype (Table 4). For systemic patients, patient assessment showed strong correlations with pain scores and joint count, similar to data for physician assessment. For polyarticular and enthesitis patients, correlation of patient global assessment with pain scores was strong. Moderate correlations were found between patient global assessment and joint count in oligoarticular and polyarticular patients.

**Table 4:** Spearman's correlations Patient Global Assessment and other outcome measures in JIA subtypes

		Systemic	Oligo-p	RF- poly	RF+ poly	Enthesitis
*Patient Global Assessment vs.*						

	pain score	-0.80	0.69	-0.74	-0.84	-0.67

	joint count	-0.73	-0.58	-0.58	-0.41	-0.35

## Discussion

The aim of this study was to determine the extent to which physician global assessment correlates with pain and joint count for JIA patients with different subtypes, using data extracted from the electronic medical record. We found the extraction process efficient, permitting a study too time consuming to perform in our center, if data were only available from paper records. Distribution of JIA subtypes was similar to that previously reported [9], except for fewer numbers of psoriatic arthritis patients, perhaps because of different ethnic distributions or shorter period of time for data collection in our study. Half of our enthesitis patients were ANA positive, possibly a result of small sample size.

Validating data was necessary to ensure data extraction yielded identical target and source data. For example, we found incorrect EMR formatting caused some data appear to be absent, when physician or patient global assessment was entered as zero. This was easily corrected, but would have been missed had there not been a validation process. When dates of disease onset and diagnosis were prior to EMR implementation, requiring data extraction from paper records, we used at least two inspections of the data. Although prospective studies eventually will not derive data from paper records, data validation from EMR sources and targets will always be necessary, if only because necessary EMR software upgrades have potential to alter data flow from source to target applications. Re-identification of de-identified data is also critical for data validation in research using data extracted from EMRs. To verify that data contents and formats have been maintained from the moment of data entry into the EMR to the time of data uploading to the data target, cross checking against data from several re-identified patients is best carried out by visual comparisons by the investigator.

In the current study, we found physician global assessment of persistent oligoarticular and rheumatoid factor negative polyarticular patients strongly correlated with joint count but moderately with patient pain scores and global assessment (Table 3). However, patient assessments showed higher correlation with pain scores than joint counts in polyarticular patients (Table 4), raising the possibility that these patients factored pain more than extent of arthritis into their global assessments. In contrast to these JIA subtypes, only for systemic JIA patients were there strong correlations between physician global assessments and all the other outcome measures tested (Table 3). This possibly reflects differences in disease characteristics in this subtype compared to others, although low joint counts in this group probably accounted for the high correlation coefficients between physician and patient assessments.

In contrast to oligoarticular and rheumatoid factor negative patients, in enthesitis patients we found a strong correlation between physician global assessment and pain score, rather than joint count. Most likely, enthesitis contributed to these correlations. A quantitative measure of enthesitis on physical examination, that might correlate with physician and patient assessments, is lacking, perhaps because of difficulties inherent in validation. Correlations between pain scores and joint counts in all JIA subtypes tended to be lower than other correlations (data not shown), possibly because pain assessment during clinic visits can be insensitive, suggested by a study using daily pain diaries [10].

Physician global assessment, while a subjective interpretation of patient status, has been used as a component of outcome scores, validated for drug studies [4] and JIA remission criteria [11]. Few investigators have explored its relationship to other outcome score components. In one study with findings similar to ours, Berntson et al found a strong relationship with joint count [12] in 312 Scandinavian patients with all JIA subtypes, excluding systemic onset. This study did not compare physician global assessment to patient global assessment or pain scores, as it addressed the role of joint size in physician assessment.

We found no differences among JIA subtypes for data distributions of physician or patient global assessments, or for joint count, perhaps because of small sample sizes and large numbers of patients with zero joint counts. Findings from this preliminary study need to be confirmed with studies of larger numbers of patients, which may identify differences among subgroups. We did not characterize whether physician assessment correlated to medication status; multi-center longitudinal studies may be better able to address this question. Finally, although it is possible that physician assessment varied, when we analyzed data from patients by physician, no such variability was found.

Ours is one of the first studies of JIA patients to use the EMR as a source of data, which can be used for quality improvement studies [13], as has been used for adults with arthritis [14]. While quality studies for children with arthritis using EMR derived data remain to be published [15], the Child Arthritis and Rheumatology Research Alliance is establishing registries for childhood rheumatic diseases, based upon widespread interest and participation of pediatric rheumatologists [16,17].

In conclusion, we have established extractable clinical data in electronic medical records for the purpose of monitoring the status of JIA patients. In this preliminary study, we found correlates of physician assessment vary with disease subtype. We anticipate adding other response measures and using extracted data to contribute to multi-center national registries.

## Competing interests

The authors declare that they have no competing interests.

## Authors' contributions

MLM contributed to study conception and design, entered data in the EMR, had full access to all data, and is responsible for data integrity and analysis. MKG contributed to study design, entered data in the EMR, and contributed to manuscript drafts and revision. JR, GL, and SM carried out implementation of the data extraction and uploading process. DW and YZ carried out all statistical analyses. All authors were involved in drafting and approving the final version of the manuscript.
